# Could Robots Help Older People with Age-Related Nutritional Problems? Opinions of Potential Users

**DOI:** 10.3390/ijerph15112535

**Published:** 2018-11-12

**Authors:** Sylwia Łukasik, Sławomir Tobis, Katarzyna Wieczorowska-Tobis, Aleksandra Suwalska

**Affiliations:** 1 Department of Human Evolutionary Biology, Institute of Anthropology, Faculty of Biology, Adam Mickiewicz University in Poznań, 61-614 Poznań, Poland; lukasik@amu.edu.pl; 2 Department of Geriatric Medicine and Gerontology, Laboratory of Occupational Therapy, Poznan University of Medical Sciences, 60-781 Poznań, Poland; stobis@ump.edu.pl; 3 Department of Palliative Medicine, Laboratory for Geriatrics, Poznan University of Medical Sciences, 61-245 Poznań, Poland; kwt@tobis.pl; 4 Department of Psychiatry, Laboratory of Neuropsychobiology, Poznan University of Medical Sciences, 60-572 Poznań, Poland

**Keywords:** assistive robotics, elderly, diet, old adults, caregivers, acceptance

## Abstract

**Background:** The rapid development of new technologies has recently raised interest in the use of assistive robots in the care of older people. The success or failure of robots’ implementation is mediated by their acceptance by users. The aim of the study was to answer the question of how both older people and caregivers perceive the possibility of using an assistive robot for nutritional support. **Methods:** Opinions of 252 respondents (126 older—aged 65 and above; 126 younger ones—aged below 65) from five European countries were collected using the Users’ Needs, Requirements, and Abilities Questionnaire. **Results:** The majority of participants positively assessed the possibility of using a robot to improve the diet of older people by giving advice on healthy eating or monitoring the amount of food consumed by the owner. An age-related difference was observed, as older people less frequently accepted the reminding of meal times or drinking liquids than younger participants. Four key categories related to the robot’s role in the diet of older people were identified: matching the needs, robot’s characteristics, ethical issues and technical/financial issues. **Conclusions:** Our preliminary research has shown a positive approach to robots in the context of their nutrition-related aspects. An indication has been made of the need to include older people and other stakeholders in the process of designing these functionalities and modeling human–robot interactions based on communication theories.

## 1. Introduction

In recent years, populations of many countries have been aging at an alarming rate. According to Eurostat data, in 2016 the share of older individuals (65 or over) in the total population of the European Union was 19.2%, whereas 10 years earlier it was only 14.9% [1]. Not only was an increase in the percentage of older people observed, but also an increase in the number of people living alone. In 2015, one-third (32.1%) of older persons were living alone [1]. The growing proportion of older people in society is due to improved healthcare and nutrition [2]. Europe is currently one of the oldest regions in the world [3]. It is estimated that the number of people aged over 65 will increase to approximately 30% by 2060 [4]. The aging of European society is currently one of the greatest social and economic challenges facing the European Union [5].

Aging of societies leads to a growing interest in lifestyle modifications that may have a beneficial effect on the aging process. One of them is a healthy diet, which contributes to the prevention of chronic diseases. It is well known that an inadequate diet is associated with an increased risk of developing numerous diseases/conditions such as hypertension, atherosclerosis, diabetes or osteoporosis [6]. Therefore, attention is paid to the pressing need for education aimed at positive lifestyle changes and promoting the principles of healthy eating [7].

Evidence of the role of diet in the cognitive functioning and the health state of older people is increasing. Finnish studies, investigating the long-term effectiveness of the Nordic diet in preventing cognitive decline, showed a positive association between diet and cognition [8]. In addition, greater adherence to the Mediterranean diet was associated with better neurocognitive test results in older individuals at risk of metabolic syndrome [9]. The findings of the Hardman team study suggested that the MIND (Mediterranean-DASH Intervention for Neurodegenerative Delay) diet was associated with substantially slowed cognitive decline with age [10]. Studies concerning the DASH (Dietary Approaches to Stop Hypertension) diet point to its effectiveness in lowering systolic and diastolic values of blood pressure [11], a beneficial effect when it comes to the risk of developing cardiovascular diseases. Furthermore, appropriate protein [12] and vitamin [13] intake is essential for maintaining optimal functioning in older people. Nonetheless, one should keep in mind the barriers to changing the eating habits of older adults. Studies conducted in Europe have shown that one of the most important and common barriers is the older people’s belief that their eating habits are healthy and require no modification. Difficulties to adopt healthier eating habits among older people can result from, among others, insufficient information, food cost or preparation [14].

Older people generally prefer to stay at home as long as possible, and many of them need support in performing daily tasks, with a special focus on the nutritional aspects. The desire of most older persons to remain in their own home (in concordance with the paradigm of ‘aging in place’) creates a need (particularly for families) to provide appropriate assistance there [15,16]. Unsurprisingly, research on informal caregiving has highlighted the intense emotional and physical burden placed on many caregivers [17]. Henceforth, there is value in approaches which minimize this burden, and using technological solutions is one of the possibilities here.

The rapid development of new technologies has recently raised interest in the use of assistive robots for the care of older people. Robots may not only help elderly people prepare meals [18] or eat them [19,20] but also remind them of their regular consumption. What is more, such devices may encourage their users to eat more healthy (wholesome) meals, as well as suggest specific recipes or prepare lists of ingredients necessary to prepare them. Assisting robots can support not only older people, but also their formal and informal carers, both in the home environment and in nursing facilities. The success or failure of the robot’s implementation is mediated by its acceptance by its users [21]. Social influence is believed to play an important role in the adoption of technology. The use of robots by older adults can be facilitated by their children or health professionals who provide necessary information, support and encourage them to use the technology [22,23,24]. Due to the wide range of potential users (including the older persons themselves, and formal and informal caregivers), a user-centered approach to the design and delivery of assistive technology must be employed [25].

The aim of the current study was to answer the question of how both older people and caregivers perceive the possibility of using an assistive robot for nutritional support. A thorough understanding of people’s needs regarding such devices can contribute to the creation of a robot meeting the expectations of both users and their caregivers. To the best of our knowledge, the use of robots in this context has not been previously analyzed.

## 2. Material and Methods

The study was carried out within the frame of the international ENRICHME project (ENabling Robot and assisted living environment for Independent Care and Health Monitoring of the Elderly). The aim of the project was to test the support of a robot for persons with mild cognitive impairment (MCI, greater cognitive decline than expected for an individual’s age and education level but does not interfere notably with activities of daily life [26]) in their living environment [27].

All subjects gave their informed consent for inclusion before they participated in the study. The study was conducted in accordance with the Declaration of Helsinki, and the protocol was approved by the Ethics Committee of Poznan University of Medical Sciences, Poland (Resolution No. 358/15). The first step in the project was to ask older people and caregivers for opinions about the required essential functionalities of robots as well as concerns about their everyday domestic use. All participants were presented with a photograph of the Kompaï robot (Robosoft, France), developed to assist seniors in their homes. It helped the examined person imagine how such a device could potentially look like. The study was conducted in person, and every participant was able to ask the interviewer questions in case of any doubts. A mixed quantitative and qualitative approach was employed, [28] which benefited from the strengths of both these methods and compensated for their weaknesses [29]. The quantitative part allowed for statistical analysis of the data whereas the qualitative part delivered insights into how the answers emerged, a better understanding and provided an in-depth analysis of the individual perspectives. This approach allowed the expansion of the perspectives that permitted a fuller treatment, description and explanation of the subject area [30].

A Users’ Needs, Requirements, and Abilities Questionnaire—UNRAQ was designed by our team on the basis of literature data regarding interactions between older people and assistive robots, and opinions of experts involved in the ENRICHME project [31].

The questionnaire included:

▪ A series of three statements about the robot’s functions, which the respondents were asked to comment on. Answers in the questionnaire were structured using the 5-point Likert scale [32]. In order to obtain a points-based score the following values were assigned to the answers: I strongly disagree—1 point; I partially disagree—2 points; I neither agree nor disagree—3 points; I partially agree—4 points and I strongly agree—5 points. The questionnaire had, in addition to the three analyzed statements, room for free remarks which constituted the qualitative part.
▪ The possibility to freely comment on the proposed solutions, add own ideas or share thoughts. The participants were asked to give free rein to their imagination and feel free to report any observations that come to mind in the context of the robot [31].

Respondents were recruited among persons who were conveniently available to participate in the study and willing to share their opinions on the use of robots to support older people living in the home environment.

The UNRAQ questionnaire was used to collect opinions about robots from 252 respondents (126 older ones—aged 65 and above and 126 younger ones, aged below 65) in five European countries, which took part in the ENRICHME project. The description of the respondents’ group is presented in Table 1.

### Data Analysis

The quantitative data were analyzed using Statistica 13 (StatSoft Polska, Kraków, Poland). The Likert scale was transformed to a 3-point-scale. Two categories of the scale (partially agreeing—4 points and strongly agreeing—5 points) were merged and re-coded into one category—‘positive responses’. The same procedure was employed to answers partially disagreeing and strongly disagreeing which were re-coded into the category of ‘negative responses’.

Chi-square test at the 0.05 level was employed for statistical significance.

The qualitative part of the study was analyzed based on the grounded theory methodology described by Strauss and Corbin [33,34,35], also called a ‘Straussian’ approach [36]. This approach consists of a set of steps, a careful execution of which is thought to ‘guarantee’ a good theory as the outcome [37]. In the Straussian approach, researchers play an active role in the process by interrogating collected data to delineate conceptual categories. It allows the researcher to predetermine the general subject of enquiry before entering the research site. An open coding and thereafter the axial and selective coding were performed by two independent researchers. All variables were discussed until a consensus was reached. On this basis, four main categories were identified, with related subcategories. One of the major advantages of the Straussian approach lies in its more structured and practically oriented method in generating grounded theory [38].

## 3. Results

### 3.1. Quantitative Analysis

The group of persons aged 65+, potentially interested in the use of a robot, included 83 women (65.9%) and 43 men (34.1%). The younger group included 87 women (69.0%) and 39 men (31.0%).

Both older and younger participants of the study positively assessed the possibility of using a robot to improve the diet of older people (by giving answers: I totally agree, or I partially agree). As many as 80.6% of the survey participants stated that *The robot should give advice on healthy eating* (77.8% of the older group vs. 83.3% of the younger group), and 71.4% stated that *The robot should monitor the amount of food and drink consumed by the owner* (69.8% of the older participants vs. 73.0% of the younger ones).

When it came to the statement *the robot should remind the older person of meal times or of drinking liquids*, the older responders expressed a positive opinion significantly less frequently than younger participants (67.5% vs. 89.2%—*p* < 0.05).

**Figure 1:**
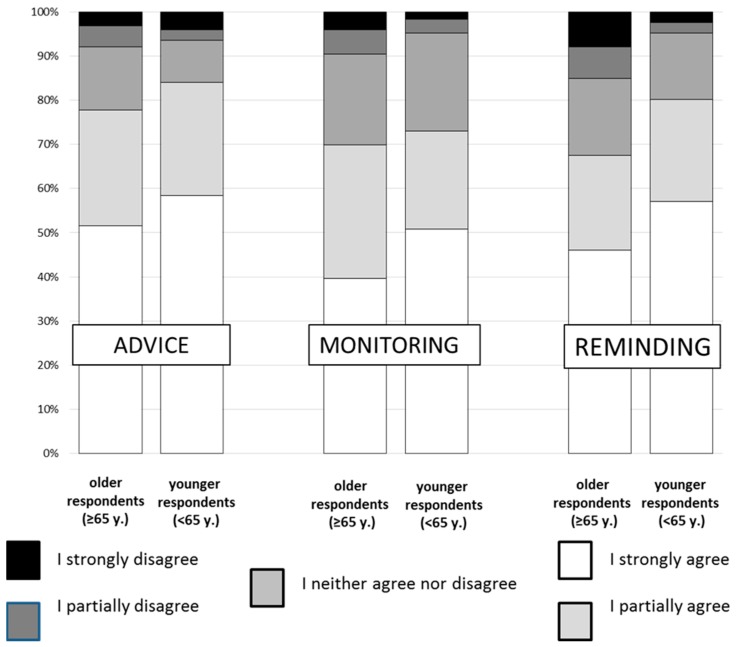
Opinions of older and younger respondents about the role of a robot in older persons’ diet. y.—years.

Detailed data on the frequency of the individual answers are presented in Figure 1. Regardless of the age group, for all three questions, the vast majority of respondents agreed with the statement on the usefulness of the assisting robot as a device supporting the nutrition of the elderly (positive answers—5 and 4).

### 3.2. Qualitative Analysis

Four key categories related to the robot’s role in the diet of older people have been identified; sub-categories have been defined in each of them (Table 2).

## 4. Discussion

The results of our preliminary study suggest that older adults are generally open to robot assistance in support of their independent living. Understanding attitudes of older people towards assistive technologies and acceptance of assistive robots is crucial to the design of both the robot’s capabilities and interactions between robots and older adults to serve their needs [39]. However, the research carried out to date has largely been about the acceptance of existing robots assisting older people [22,40] rather than about the expectations of users regarding the robot (especially the ones related to nutrition, as is the case with our research). The latter type of research is particularly important because the expectations of potential users does not necessarily match the expectations of those responsible for the design and construction of such robots [41]. Therefore, knowing the specific needs of potential users is essential to ensuring that they will not only want to benefit from the help of an assistive robot, but will also feel good in its presence [42].

Previous research examining the acceptance of a robot by potential users has shown that preparing meals and cooking belong to those activities in which the respondents strongly favor human help [39]. Cooking and reheating meals were considered a necessary function by the caregivers rather than older people [43]. However, an involvement of the robot in other nutrition-related activities was not assessed in these studies. Our study revealed a positive attitude of respondents to the robot in terms of these particular activities. For older people who are more sensitive to food deficiencies than younger individuals, regular eating and drinking of fluids are especially important. Monitoring in this area would be particularly useful for people with mild cognitive impairment, since dehydration or deficits of nutrients may lead to further deterioration of their cognitive functions [44]. In this context, it is important to emphasize the fact that caregivers are more likely to accept this function than the older people themselves. It is, therefore, necessary to educate older adults about the potential consequences of malnutrition and dehydration in order to increase their awareness of the importance of regularity in meals and fluid intake. However, it must be remembered that the older people interviewed in our study were independent and might not have related this function to their own needs. Older persons point out that unnecessary help can impair their self-reliance. Our results in this regard are in line with previous research showing that older people actually expect help from a robot instead of being replaced by it in the activities of daily living [45].

A high level of acceptance was observed with the functions related to giving tips on healthy eating. A robot that assists older persons in preparing meals has already been developed [18]. It uses a tablet to present a list of recipes to the user, then helps locate the specific ingredients in the kitchen and shows a short film presenting the stages of making a given meal. This robot, however, does not give tips on healthy eating and does not advise on what foods should be avoided in the event of dietary restrictions due to allergies or illnesses (for example, diabetes). The results of our survey showed that older people believed that a robot could provide dietary advice. In our opinion, this functionality should be added to robots being designed. The respondents emphasized the importance of not transmitting observational data by the robot to other people without the user’s acceptance. Older people fear losing not only privacy but also control over their lives [46], henceforth the question arises who should have access to the information obtained and archived by the robot and who could decide on the access. Some earlier work on assisting robots has also raised similar ethical concerns (cf. [46,47]). Ethical and practical issues must be taken into account when designing the functions and the technical environment of the robot. The participants pointed out that they did not want to be spied on by the robot, so it would be necessary to individualize the development of the robot’s functions to avoid creating a feeling of ‘controlling’ the life of the older person [48].

The respondents indicated that an assisting robot should be programmed in a way that its presence is not too burdensome for them (for example, by too often reminding them of the need to eat or interfering at the wrong time). Monitoring robots should be flexible, able to recognize situations, interpret them, and make decisions in a specific situation so as to limit contacts with older persons to the necessary minimum [49].

An important observation is the fact that older people accepted the meals and drinks reminding function less often than younger persons while the percentage of positive answers regarding diet advice and monitoring did not differ significantly between groups. The human-robot interaction may be analyzed in terms of the politeness theory in which all members of society have “face”—the public self-image that every member wants to claim for themselves and tries to protect [50,51]. Offering someone help can be a face-threatening act [52], this may also apply to the help provided by the robot in our survey. Monitoring of a person interferes with their claim to space and freedom from distraction; giving advice creates pressure on the person to either perform or not perform the act [50]. Reminding may be perceived as giving orders, which is a very threatening act to a listener’s autonomy [50,51]. The results of our study suggest that older persons may perceive the use of a robot as a threat to their autonomy and self-esteem, and confirm the findings of the study by Torrey et al. that the communication strategy should be integrated into the delivery of advice by a robot [51]. Self-esteem or “face” of an older user has to be taken into consideration in the process of the design and development of robots, as a robot coexisting with humans must be able not only to successfully perform physical tasks but also to interact with humans in a socially appropriate manner, involving adequate language [53]. Reminding of meal or drink times was addressed by Korchut et al. [54]. Their results showed that—according to older people with cognitive impairment—this functionality is not necessary in assistive robots whereas, in the opinions of caregivers and medical staff, the robots should have the ability to perform this task. This discrepancy may be due to the fact that for older adults it is difficult to agree that they must be reminded about the simplest and basic human needs. This can be perceived as a way of admitting a defeat [55].

It should also be noted that people over 65 years, when completing the questionnaire, tend to think about what immediate impact a robot would have on their own living situation, whereas those under 65 years are rather considering an impact of the robot on persons older than 65 years of age (so not themselves), hence they commonly assess acceptability of certain statements not having personally experienced loss of independence.

The limitation of our research is its cross-sectional nature, which does not allow observing changes in attitudes caused by gaining additional information. It would be interesting to conduct longitudinal studies, as this may change not only the perception of robots but also the patterns of their acceptance [56]. It can be assumed that with the increase of availability of assisting robots on the market and their greater choice (in terms of models and functions), and hence the fall in prices of these devices, the level of acceptance and demand for them will change, just like in the case of other electronic devices. The relatively small number of respondents from several European countries makes it difficult to make reliable comparisons of international data. On the other hand, the multi-national nature of our study undoubtedly constitutes its advantage, ensuring the diversity of the studied group.

## 5. Conclusions

The accurate evaluation of the individual needs and preferences of potential users followed by matching the functionality of the robot to them may ensure greater acceptance and significantly improve the quality of life of end-users. Our research has shown a positive approach to robots in the context of their nutrition-related aspects. An indication has been made of the need to include older people and other stakeholders in the process of designing these functionalities and in modeling human–robot interactions based on communication theories.

## Figures and Tables

**Table 1:** Characteristics of the studied group.

	Older Respondents (Number of Females) Age (Years) Mean ± SD; Median	Younger Respondents (Number of Females) Age (Years) Mean ± SD; Median
France	13 (5) 70.5 ± 4.7; 69.0	0
Greece	37 (26) 74.8 ± 6.8; 74.0	37 (20) 43.0 ± 15.2; 42.0
Italy	32 (22) 81.5 ± 9.7; 84.0	19 (13) 46.3 ± 10.5; 46.0
Poland	35 (23) 71.4 ± 5.6; 70.0	56 (42) 31.8 ± 8.8; 28.0
Great Britain	9 (7) 77.1 ± 9.2; 75.0	14 (10) 42.5 ± 11.8; 43.5
All respondents	126 (83) (75.3 ± 8.4; 74.0)	126 (85) (38.5 ± 13.0; 35.5)

Mean age of the older group was 75.3 ± 8.4 (range 65–100; median 74) and of the younger one—38.5 ± 13.0 (range 20–64; median 35.5).

**Table 2:** Qualitative analysis of respondent statements.

Categories	Sub-Categories	Examples
Matching the needs	Encouragement to eat and drink	“Sometimes I really eat too little. The robot could help me”
	Optionality	“These functionalities should be optional: ‘on’ to whom it is useful and ‘off’ to whom it is useless”
	State of health	“It would help if I take an extra biscuit… I should not because I have high blood sugar”
Robot’s characteristics	Physical traits of the robot	“The robot has no arms for serving food”
	Functionalities	“The robot could suggest dinner ideas and tell me what to cook so that I don’t eat the same things all the time”
	Interaction with the user	“The robot should remind about meals and drinks with kind manners”
Ethical issues	Privacy	“It is OK if the robot controls me and tells me to avoid eating too much…but it must not tell anybody else. He should not make the gossiper, not to spy”
	Issue of robots replacing humans	“A robot will never replace a visit at a dietician”
Technical/financial issues	Unreliability of technology	“And what if the robot breaks down?”
	Costs of the robot	“Who will be able to afford such a robot?”
